# Une fente orbito-faciale bilatérale

**DOI:** 10.11604/pamj.2014.18.67.3197

**Published:** 2014-05-19

**Authors:** Bouchra Alami, Rachid Sekhsoukh

**Affiliations:** 1 Service d'Ophtalmologie, Centre Hospitalier Mohammed VI, Faculté de médecine et pharmacie, Université Mohammed premier, 60 000, Oujda, Maroc

**Keywords:** Fente orbito-faciale, malformation, Orbito-facial cleft, malformation

## Image en medicine

Les fentes faciales sont des malformations rares résultant d'une absence totale ou partielle de fusion des bourgeons faciaux. La classification des fentes faciales a été mise au point par le pionnier de la chirurgie crânio-faciale, Paul TESSIER, en 1976, et prend l'orbite pour référence anatomique. Elle est cotée de 0 à 14 et concerne en général les tissus mous et massifs osseux. Les fentes sont numérotées de 0 à 7 pour l'hémisphère sud et de 8 à 14 pour l'hémisphère nord; soit quinze fentes correspondants à des axes constants. Les fentes faciales touchent l'orbite osseuse, paupières, les lèvres ainsi que les maxillaires. Elles portent un numéro à un chiffre. L'image montre l'existence d'une fente orbito-faciale bilatérale 4 de Tessier, chez un prématuré, elle traverse presque verticalement le segment lacrymal de la paupière inférieure, le rebord infra-orbitaire, le plancher de l'orbite. En bas, elle passe par le sinus maxillaire, avant de rejoindre la lèvre entre filtrum et commissure. Les fentes crânio-faciales sont des fentes qui se prolongent vers le crâne. Elles portent un numéro à deux chiffres. Les fentes orbito-faciales sont centrées sur l'orbite et doivent de ce fait être connu par l'ophtalmologiste afin de permettre une prise en charge médicale et chirurgicale optimale.

**Figure 1:**
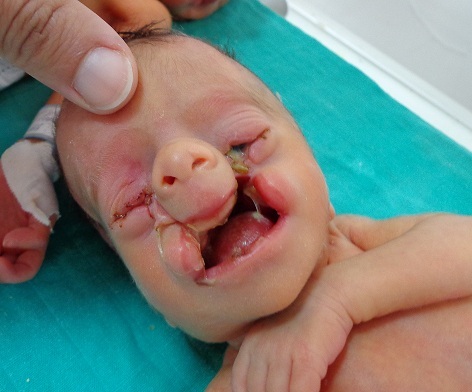
Fente orbito-faciale bilatérale 4 de Tessier chez un prématuré: la fente traverse presque verticalement le segment lacrymal de la paupière inférieure, le rebord intra-orbitaire, le plancher de l'orbite. En bas, elle passe par le sinus maxillaire et joint la lèvre entre filtrum et commissure

